# Emerging SARS-CoV-2 Resistance After Antiviral Treatment

**DOI:** 10.1001/jamanetworkopen.2024.35431

**Published:** 2024-09-25

**Authors:** Trevor J. Tamura, Manish C. Choudhary, Rinki Deo, Fizah Yousuf, Anadela Navarrete Gomez, Gregory E. Edelstein, Julie Boucau, Owen T. Glover, Mamadou Barry, Rebecca F. Gilbert, Zahra Reynolds, Yijia Li, Dessie Tien, Tammy D. Vyas, Eliza Passell, Karry Su, Sarah Drapkin, Emory G. Abar, Yumeko Kawano, Jeffrey A. Sparks, Zachary S. Wallace, Jatin M. Vyas, Robert W. Shafer, Mark J. Siedner, Amy K. Barczak, Jacob E. Lemieux, Jonathan Z. Li

**Affiliations:** 1 Brigham and Women’s Hospital, Boston, Massachusetts; 2 Harvard Medical School, Boston, Massachusetts; 3 Ragon Institute of Massachusetts General Hospital, MIT and Harvard, Cambridge; 4 Massachusetts General Hospital, Boston; 5 University of Pittsburgh Medical Center, Pittsburgh, Pennsylvania; 6 Broad Institute, Cambridge, Massachusetts; 7 Stanford University School of Medicine, Palo Alto, California

## Abstract

**Importance:**

Previous studies have identified mutations in SARS-CoV-2 strains that confer resistance to nirmatrelvir, yet how often this resistance arises and its association with posttreatment virologic rebound is not well understood.

**Objective:**

To examine the prevalence of emergent antiviral resistance after nirmatrelvir treatment and its association with virologic rebound.

**Design, Setting, and Participants:**

This cohort study enrolled outpatient adults with acute COVID-19 infection from May 2021 to October 2023. Participants were divided into those who received antiviral therapy and those who did not. The study was conducted at a multicenter health care system in Boston, Massachusetts.

**Exposure:**

Treatment regimen, including none, nirmatrelvir, and remdesivir.

**Main Outcomes and Measures:**

The primary outcome was emergent SARS-CoV-2 antiviral resistance, defined as the detection of antiviral resistance mutations, which were not present at baseline, were previously associated with decreased antiviral efficacy, and emerged during or after completion of a participant’s treatment. Next-generation sequencing was used to detect low frequency mutations down to 1% of the total viral population.

**Results:**

Overall, 156 participants (114 female [73.1%]; median [IQR] age, 56 [38-69] years) were included. Compared with 63 untreated individuals, the 79 who received nirmatrelvir were older and more commonly immunosuppressed. After sequencing viral RNA from participants’ anterior nasal swabs, nirmatrelvir resistance mutations were detected in 9 individuals who received nirmatrelvir (11.4%) compared with 2 of those who did not (3.2%) (*P* = .09). Among the individuals treated with nirmatrelvir, those who were immunosuppressed had the highest frequency of resistance emergence (5 of 22 [22.7%]), significantly greater than untreated individuals (2 of 63 [3.1%]) (*P* = .01). Similar rates of nirmatrelvir resistance were found in those who had virologic rebound (3 of 23 [13.0%]) vs those who did not (6 of 56 [10.7%]) (*P* = .86). Most of these mutations (10 of 11 [90.9%]) were detected at low frequencies (<20% of viral population) and reverted to the wild type at subsequent time points. Emerging remdesivir resistance mutations were only detected in immunosuppressed individuals (2 of 14 [14.3%]) but were similarly low frequency and transient. Global Initiative on Sharing All Influenza Data analysis showed no evidence of increased nirmatrelvir resistance in the United States after the authorization of nirmatrelvir.

**Conclusions and Relevance:**

In this cohort study of 156 participants, treatment-emergent nirmatrelvir resistance mutations were commonly detected, especially in individuals who were immunosuppressed. However, these mutations were generally present at low frequencies and were transient in nature, suggesting a low risk for the spread of nirmatrelvir resistance in the community with the current variants and drug usage patterns.

## Key Points

**Question:**

Is there an increased frequency of SARS-CoV-2 antiviral resistance in individuals receiving antiviral therapy?

**Findings:**

In this cohort study with 156 participants, nirmatrelvir resistance mutations were detected more often in individuals who were treated with nirmatrelvir, especially those who were immunosuppressed, compared with untreated individuals. However, all mutations were present at relatively low frequencies, appeared transiently, and were unlikely contributors to instances of virologic rebound.

**Meaning:**

In this study, the emergence of SARS-CoV-2 strains with resistance to nirmatrelvir after treatment was rare, irrespective of virologic rebound.

## Teaser

This cohort study examines whether treatment with nirmatrelvir and remdesivir is associated with emergent antiviral resistance in SARS-CoV-2 strains.

## Introduction

Nirmatrelvir and remdesivir are SARS-CoV-2 antivirals recommended for use in mild to moderate COVID-19 to reduce risk of progression to severe disease and hospitalization in high-risk individuals.^1,2,3^ Nirmatrelvir, the active component of nirmatrelvir-ritonavir, inhibits the main protease (Mpro) of SARS-CoV-2 and blocks the cleavage of the viral polyprotein precursors.^1^ Remdesivir, a prodrug of the adenine nucleoside analogue, GS-441524, inhibits the RNA-dependent RNA polymerase (RdRp) of SARS-CoV-2 and blocks viral RNA synthesis.^3^

The risk of treatment-emergent drug resistance after SARS-CoV-2 antiviral therapy remains unclear. While several in vitro studies have reported on either naturally occurring or dose-dependent emergent resistance to these antivirals, detection of in vivo resistance has been relatively rare.^1,3,4,5,6,7,8,9,10,11^ In the clinical trials for nirmatrelvir, EPIC-HR/SR, nirmatrelvir resistance emerged in 0.3% of participants (3 of 907).^1^ However, the prevalence of nirmatrelvir resistance in clinical settings remains unknown. In addition, prior studies mainly used consensus sequencing to identify resistance, which only captures majority variants within the viral population and is unable to detect low frequency resistance mutations that could contribute to a resistance phenotype.

An additional concern is the possibility of an association between posttreatment virologic rebound and emergence of antiviral resistance. While virologic rebound has been observed in a subset of patients following nirmatrelvir treatment, consensus sequencing in these studies did not identify nirmatrelvir resistance mutations during rebound.^12,13,14^ By contrast, a previous study of monoclonal antibody therapy demonstrated that deep sequencing can identify low frequency resistance mutations that subsequently become the dominant variant in the viral population and contribute to virologic rebound, highlighting the need for the same type of surveillance in patients who receive antiviral treatment.^15^ In the present study, we focused on the in vivo emergence of mutations that confer resistance to nirmatrelvir and remdesivir, aiming to assess mutation prevalence at low frequencies and any association with posttreatment virologic rebound.

## Methods

### Study Design, Sample Collection, and Virologic Rebound Definition

The Post-Vaccination Viral Characteristics Study (POSITIVES) is an ongoing prospective cohort study of individuals with acute COVID-19 within the Mass General Brigham (Boston, Massachusetts) health care system. This analysis includes a total of 256 participants (237 in the nirmatrelvir cohort and 19 in the remdesivir cohort) from the POSITIVES study who were enrolled between May 2021 and October 2023 (eFigures 1 and 2 in Supplement 1). Of the 237 in the nirmatrelvir cohort, 95 individuals were excluded based on receiving nonnirmatrelvir therapies, time of enrollment, number of samples collected, and the availability of cDNA and detectable viral load samples for sequencing (eFigure 1 in Supplement 1). Of the 19 in the remdesivir cohort, 5 individuals were excluded based on the availability of cDNA and detectable viral load samples for sequencing (eFigure 2 in Supplement 1).

Anterior nasal (AN) swab samples are collected 3 times a week for the first 2 weeks following study enrollment and then weekly thereafter until participants have persistently undetectable viral loads (eMethods in Supplement 1). Participant demographics were taken from the EPIC database, where some of their information, like race and ethnicity, were self-reported to their clinician when they registered into the Mass General Brigham system. Race and ethnicity were gathered because this multi-institutional collaborative project involved data collection for several other research efforts within our collaboration, including a study that focuses directly on how race and ethnicity affect SARS-CoV-2 viral clearance and symptom improvement. A full list of all race and ethnicity options in the database are found in eTables 1 and 2 in Supplement 1. For this analysis, racial groups were collapsed into Black or African American, White, and other or unknown (including American Indian or Alaska Native, Asian, and Native Hawaiian or Other Pacific Islander), and ethnic groups were collapsed in Hispanic or Latino, non-Hispanic or non-Latino, and other or unknown.

In this cohort, we define virologic rebound either by (1) a negative SARS-CoV-2 viral culture result followed by a positive result or (2) a nadir viral load less than 4.0 log_10_ RNA copies/mL followed by an increase in viral load of at least 1.0 log_10_ RNA copies/mL for 2 consecutive time points greater than 4.0 log_10_ RNA copies/mL. Additional study details are outlined elsewhere.^12,13^

### Ethical Considerations

All study participants provided verbal informed consent. Written consent was waived by the review committee based on the need to obtain consent for a minimal risk study during the acute phase of COVID-19 infection. The study procedures were approved by Institutional Review Board and the Institutional Biosafety Committee at Mass General Brigham. This study followed the Strengthening the Reporting of Observational Studies in Epidemiology (STROBE) reporting guideline.

### SARS-CoV-2 Viral Load Testing and Target Gene Next-Generation Sequencing

Viral RNA extraction and viral load testing were performed as previously described.^16^ For the target gene next-generation sequencing (NGS), 250 μL of an AN swab sample was used for RNA extraction via the Trizol-LS reagent, and cDNA was synthesized using Superscript IV reverse transcriptase (Invitrogen) following the manufacturer’s instructions and as described elsewhere.^15^

The *nsp5* gene, encoding Mpro, and the *nsp12* gene, encoding RdRp, were amplified with a nested polymerase chain reaction (PCR) approach, using in-house designed primers. NGS was performed using the Illumina MiSeq platform. Raw sequencing data were analyzed using Stanford University’s Coronavirus Antiviral & Resistance Database.^17^ Initial alignment of input FASTQ sequences to the Wuhan-Hu-1 reference was performed using MiniMap2 (version 2.22) within the CodFreq pipeline.^18^ The resulting aligned SAM file from MiniMap2 was then converted to a CodFreq file using an in-house Python script leveraging the PySam library (version 0.18.0) and subsequently subjected to further analysis with CoV-RDB.

The accuracy of the sequencing results was confirmed with a control library constructed from several SARS-CoV-2 variant-specific Mpro sequences mixed at known concentrations that resulted in a variety of viral frequencies, ranging from 68.4% to 0.1%.^15,19^ The control library was run with every PCR and sequencing run, providing a set of expected frequencies to use in our sequencing analysis. Our mutation detection had a false positive rate of 0.11% of the total viral population, defined as the false positive rate ±3 SDs. Furthermore, we observed strong correlations between the expected and observed frequencies of the mutations in the control library, which allowed us to set a limit of detection of 1%, above which we could confidently differentiate low frequency mutations from technical artifacts that may have occurred during the PCR and sequencing processes. Amino acid variations were identified at the codon level via Perl code and utilized for resistance interpretation with a limit of detection set at 1%, which was determined from the control library. Mutations detected through NGS at less than 20% of the viral population were classified as low frequency variants, as they would predominantly elude detection by traditional Sanger and consensus sequencing methods. A minimum average sequencing coverage of 500 × per sample was mandated for the calling of SARS-CoV-2 variants. At the codon level, amino acid variants were called at a threshold based on viral load: 1% for 3.0 log_10_ RNA copies/mL or greater and 10% for less than 3.0 log_10_ RNA copies/mL.

### Mutation Detection

Emergent resistance was defined as any antiviral resistance mutation of interest that arose during or after completion of antiviral treatment for treated individuals or at any time during observation for untreated individuals. Nirmatrelvir resistance mutations of interest were defined as the 51 mutations previously reported from in vitro and in vivo studies to confer at least 2.5-fold reduced susceptibility to nirmatrelvir as compiled in Stanford University’s Coronavirus Antiviral & Resistance Database (last checked May 1, 2024).^20^ Remdesivir resistance mutations of interest were defined as the 13 mutations associated with at least 2.5-fold reduced susceptibility to remdesivir in the same database (last checked May 1, 2024).^21^ The full list of nirmatrelvir and remdesivir resistance mutations of interest are included in eTables 3 and 4 in Supplement 1.

### GISAID Database

To assess whether there is evidence of increasing nirmatrelvir resistance in the community, we calculated the monthly prevalence of nirmatrelvir resistance mutations in the Global Initiative on Sharing All Influenza Data (GISAID) database of US SARS-CoV-2 sequences before and after the Food and Drug Administration’s (FDA) Emergency Use Authorization (EUA) was approved for nirmatrelvir in December 2021.^22^ For this analysis, we counted the total number of sequences and the variant sequences with our nirmatrelvir resistance mutations of interest across all lineages, excluding low coverage sequences, available in the United States monthly from January 2020 to February 2024.

### Statistical Analysis

To calculate significance, a 2-sided Boschloo test was used on categorical data and odds ratios (ORs) were used for demographic data. Figures were produced via Python version 3.8.11 (Python Software Foundation) and Graphpad Prism version 10.2 (Insight Partners).

## Results

In this cohort study of 156 individuals with acute COVID-19, compared with the 63 untreated participants, the 79 participants treated with nirmatrelvir were older (median [IQR] age 45 [33-62] years vs 62 [51-71] years; OR, 1.52; 95% CI, 1.22-1.88), more commonly immunosuppressed (7 [11.1%] vs 22 [27.8%]; OR, 3.46; 95% CI, 1.19-10.05), received more COVID-19 vaccinations (median [IQR], 3 [3-4] doses vs 4 [3.5] doses; OR, 1.23; 95% CI, 1.00-1.49), had a shorter number of days since their last COVID-19 vaccine (median [IQR] 224; [163-325] days vs 155 [83-248] days; OR, 0.997; 95% CI, 0.994-0.999), and experienced more frequent virologic rebound (3 [4.8%] vs 23 [29.1%]; OR, 6.46; 95% CI, 1.90-23.24) (Table). These 2 groups had similar clinical and demographic characteristics, such as sex (female: 49 [77.8%] vs 59 [74.7%]; OR, 0.84; 95% CI, 0.39-1.84), race (Black or African American: 6 [9.5%] vs 10 [12.7%]; OR, 1.26; 95% CI, 0.42-3.78; White: 43 [68.3%] vs 57 [72.2%]), and SARS-CoV-2 subvariant (BA.2: 7 [11.1%] vs 9 [11.4%]; BA.5: 19 [30.2%] vs 21 [26.6%]; OR, 0.61; 95% CI, 0.17-2.16; XBB: 20 [31.7%] vs 30 [38.0%]; OR, 0.76; 95% CI, 0.22-2.63). Additionally, none of the individuals in the nirmatrelvir-treated and untreated groups received any other COVID-19 therapies (Table). The cohort characteristics for the 14 participants treated with remdesivir are listed as well, but no comparisons were made with the untreated group, as they likely would be underpowered given the small group size of those who received remdesivir. One characteristic of note is that 3 of these participants (21.4%) received monoclonal antibody treatment in addition to remdesivir (eTable 5 in Supplement 1).

**Table.** Demographic Characteristics of Participants in This Subset of the POSITIVES Cohort

Characteristic	Participants, No. (%)		OR (95% CI)
	Untreated (n = 63)	Received nirmatrelivr (n = 79)	
Age, median (IQR), y	45 (33-62)	62 (51-71)	1.52 (1.22-1.88)^a^
Sex			
Female	49 (77.8)	59 (74.7)	0.84 (0.39-1.84)
Male	14 (22.2)	20 (25.3)	1 [Reference]
Race			
Black or African American	6 (9.5)	10 (12.7)	1.26 (0.42-3.78)
White	43 (68.3)	57 (72.2)	1 [Reference]
Other or unknown^b^	14 (22.2)	12 (15.2)	0.65 (0.27-1.54)
Ethnicity			
Hispanic or Latino	4 (6.3)	6 (7.6)	1 [Reference]
Non-Hispanic or non-Latino	49 (77.8)	67 (84.8)	0.91 (0.24-3.40)
Other or unknown	10 (15.9)	6 (7.6)	0.40 (0.08-2.02)
COVID-19 vaccines, median (IQR), No.	3 (3-4)	4 (3-5)	1.23 (1.00-1.49)
Time since last vaccine, median (IQR), d	224 (163-325)	155 (83-248)	0.997 (0.994-0.999)
mAb use	0	0	NA
Immunosuppression^c^			
Absent	56 (88.9)	57 (72.2)	1 [Reference]
Present	7 (11.1)	22 (27.8)	3.46 (1.19-10.05)
SARS-CoV-2 variant			
BA.2^d^	7 (11.1)	9 (11.4)	1 [Reference]
BA.5^e^	19 (30.2)	21 (26.6)	0.61 (0.17-2.16)
XBB^f^	20 (31.7)	30 (38.0)	0.76 (0.22-2.63)
Other	15 (23.8)	13 (16.4)	0.56 (0.13-2.32)
Not yet sequenced	2 (3.2)	6 (7.6)	1.67 (0.13-20.58)
Virologic rebound			
Absent	60 (95.2)	56 (70.9)	1 [Reference]
Present	3 (4.8)	23 (29.1)	6.46 (1.80-23.24)

Abbreviations: mAb, monoclonal antibody; NA, not applicable; OR, odds ratio.

^a^ OR of age per 10 years between untreated and nirmatrelvir-treated groups.

^b^ Other or unknown includes American Indian or Alaska Native, Asian, and Native Hawaiian or Other Pacific Islander individuals.

^c^ Immunosuppression was determined by clinicians who conducted a formal medical record review in any participant who had an immunosuppressing condition or was taking an immunosuppressing medication.

^d^ BA.2 includes all BA.2 subvariants.

^e^ BA.5 includes all BA.5 subvariants.

^f^ XBB includes all XBB subvariants.

**Figure 1.**
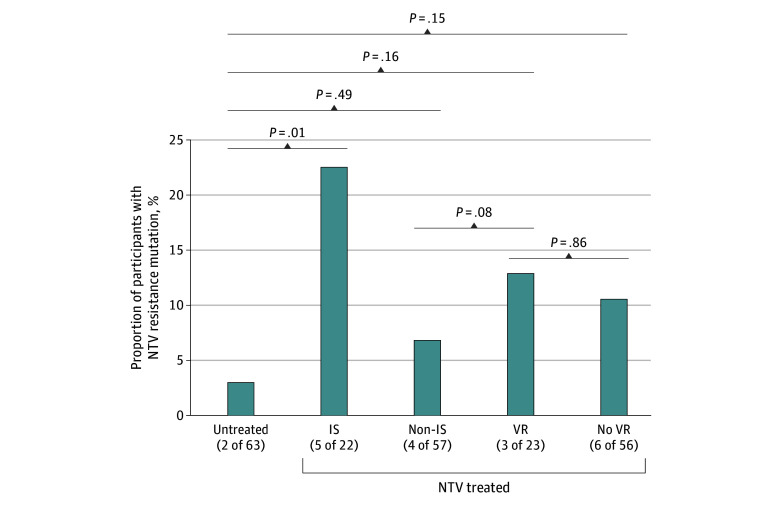
Prevalence of Emergent Nirmatrelvir Resistance Mutations in Untreated and Nirmatrelvir-Treated Individuals Individuals treated with nirmatrelvir were also separated by immunosuppression (IS) and virologic rebound (VR) status. *P* values were calculated using a 2-sided Boschloo test.

Numerically, more emergent nirmatrelvir resistance mutations were detected in the *nsp5* gene from nirmatrelvir-treated individuals (9 [11.4%]) than untreated individuals (2 [3.2%]), although this comparison was not statistically significant (*P* = .09) (eFigure 3 in Supplement 1). Within the nirmatrelvir-treated group, immunosuppressed individuals had a higher frequency of resistance-associated mutation emergence (5 of 22 [22.7%]) compared with treated, nonimmunosuppressed individuals (4 of 57 [7.0%]), although this difference was not statistically significant (*P* = .08). Immunosuppresed individuals treated with nirmatrelvir had significantly higher resistance-associated mutation emergence compared with untreated individuals (18 of 158 [11.4%] vs 2 of 63 [3.1%]) (*P* = .01) (Figure 1). The 5 immunosuppressed individuals who received nirmatrelvir and had an emergent resistance mutation were all classified as having nonsevere immunosuppression, using the definition previously described (eTable 6 in Supplement 1).^23^ Additionally, among those who received nirmatrelvir treatment, similar rates of nirmatrelvir resistance–associated mutations were observed in those who had virologic rebound (3 of 23 [13.0%]) compared with those who did not (6 of 56 [10.7%]) (*P* = .86).

**Figure 2.**
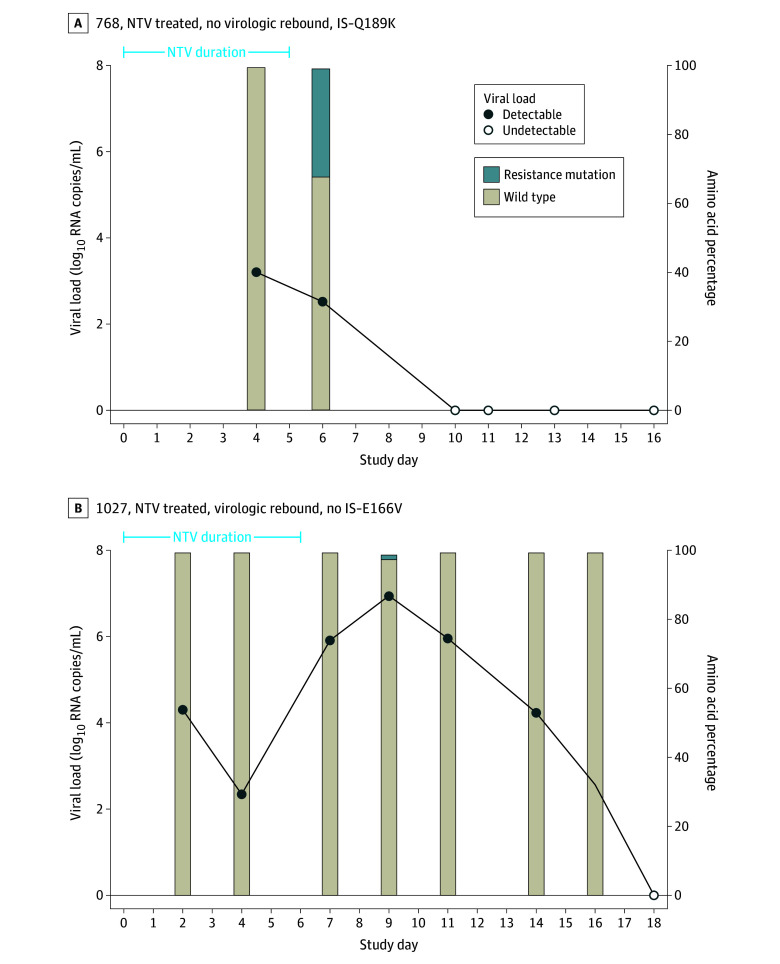
Viral Load and Mutational Landscape Graphs for Participants With an Emergent Nirmatrelvir Resistance Mutation A, An individual with the highest frequency mutation observed. B, An individual with virologic rebound. IS indicates immunosuppressed.

We identified 6 emergent nirmatrelvir resistance mutations in the nirmatrelvir treated group previously reported to confer at least 2.5-fold reduction in antiviral susceptibility (G138S, E166V, H172Y, Q189K, P252L, and V297A) and 1 in the untreated group (Q189K). Most nirmatrelvir resistance mutations (10 of 11 [90.9%]) were detected at low frequencies within a participant’s viral population (<20%) (eFigure 4 in Supplement 1). One participant had a detectable emergent nirmatrelvir resistance mutation present at 31.4% of the viral population 1 day after the end of treatment, although nasal RNA levels became undetectable at the next specimen collection 4 days later and remained suppressed for the duration of observation (Figure 2A). For the 3 nirmatrelvir-treated participants who had an emergent nirmatrelvir resistance mutation and posttreatment virologic rebound, 2 resistance mutations (66.7%) emerged before the participant’s viral load started to rebound and the other emerged at the peak viral load of the rebounding course (Figure 2B; eFigure 5 in Supplement 1). However, all mutations were present at low frequencies and were transient in nature, reverting to the wild type at time points after initial detection (Figure 2; eFigure 5 in Supplement 1).

In the remdesivir-treated group, 3 emergent remdesivir resistance mutations (N198S, V792I, and M794I) were detected in the *nsp12* gene of 2 of 14 individuals (14.3%) (eFigure 6 in Supplement 1). These 2 individuals were classified as having severe immunosuppression, one with metastatic Merkel cell carcinoma and the other with Mantle cell lymphoma (eTable 6 in Supplement 1). Similar to the emergent nirmatrelvir resistance mutations, these emergent remdesivir resistance–associated mutations were present at low frequencies and were transient, reverting to the wild type after observation at a single time point.

**Figure 3.**
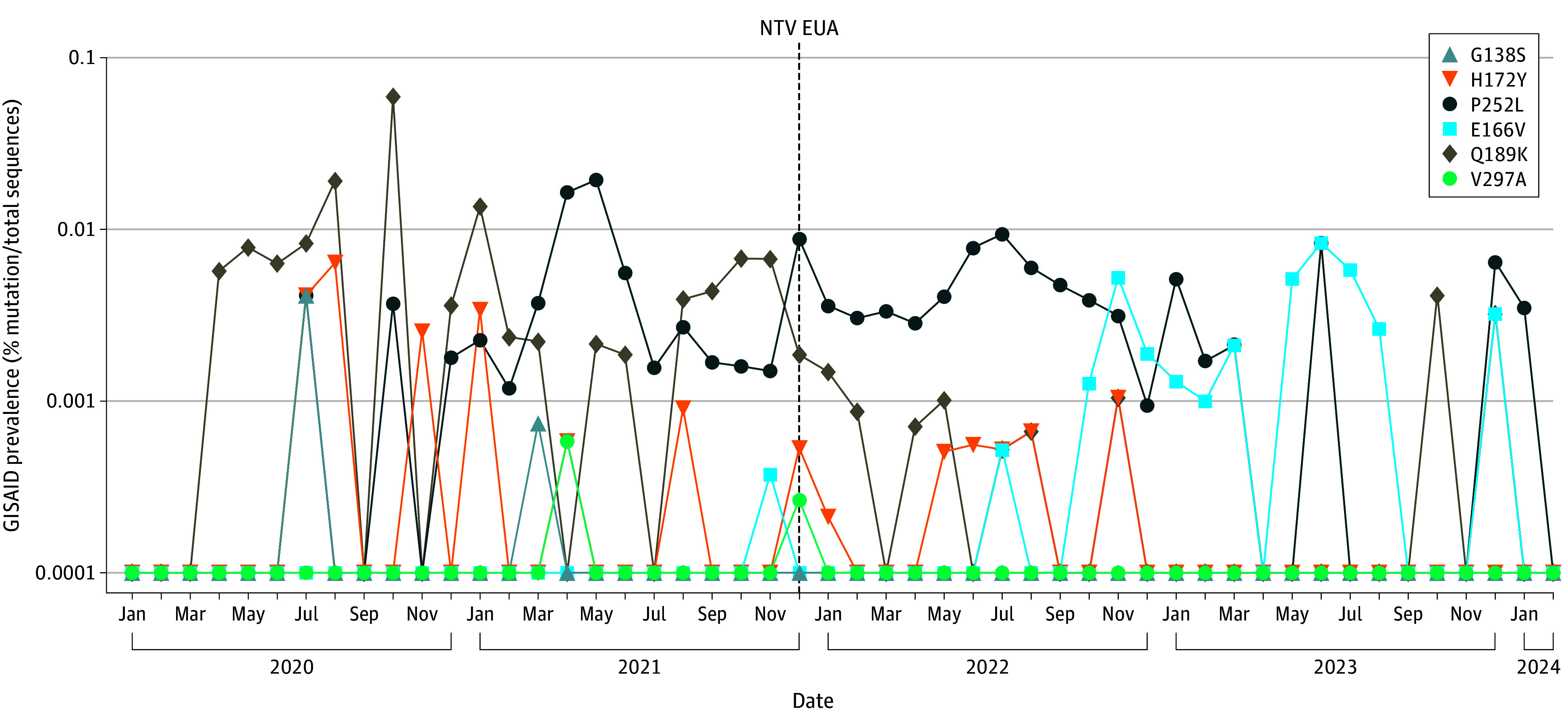
Nirmatrelvir Resistance Mutation Prevalence Compiled From Global Initiative on Sharing All Influenza Data (GISAID) SARS-CoV-2 Sequences in the United States SARS-CoV-2 sequences were downloaded from GISAID between January 2020 and February 2024. Prevalence was calculated by dividing the number of mutated variants of interest by the total number of sequences per month. The dashed line at December 2021 indicates the Food and Drug Administration’s initial Emergency Use Authorization (EUA) for nirmatrelvir.

Given the detection of nirmatrelvir resistance in our cohort and concern about its spread into the community, we assessed the frequency of resistance mutations in GISAID before and after the FDA’s EUA for nirmatrelvir in the United States. We observed a slight increase in the prevalence of the E166V mutation, from 1 of 2 575 229 sequences (0.00004%) harboring this mutation in the 24 months before the EUA to 16 of 2 487 821 sequences (0.0006%) in the 26 months after the EUA (Figure 3). However, the monthly peak E166V prevalence in the United States in June 2023 was only 1 in 10 000 sequences and was not sustained (Figure 3). Overall, there was no sustained increase in the frequency of nirmatrelvir resistance mutations in US sequences from GISAID after the FDA EUA of nirmatrelvir.

## Discussion

In this analysis of a prospective cohort of individuals with acute COVID-19, we performed a sensitive NGS approach to assess the prevalence of emergent antiviral resistance in patients in the POSITIVES study receiving either nirmatrelvir, remdesivir, or no antiviral therapy and their association with posttreatment virologic rebound. Specifically, we found that mutations associated with nirmatrelvir resistance were more commonly identified in individuals treated with nirmatrelvir who were immunosuppressed, as has been previously seen in case reports.^24,25^ However, the detected resistance mutations were mainly present at minority frequencies, were transient in nature, and were not more prevalent in individuals who experienced virologic rebound. Additionally, in combination with a GISAID analysis showing no increased prevalence of nirmatrelvir resistance in the United States over time, these data suggest a low risk of significant drug resistance with current variants and antiviral drug usage patterns.

Emergent SARS-CoV-2 antiviral resistance has been reported in in vitro viral passage studies in cell culture at different doses of either nirmatrelvir or remdesivir.^1,3,4,8,10,11^ Additionally, there have been case reports of emergent resistance in patients, such as the study published by Hirotsu et al,^25^ describing a man aged 65 years with immunosuppression and treated with multiple, prolonged courses of antibody and antiviral therapies, who developed resistance to nirmatrelvir, sotrovimab, and remdesivir. In the phase 3 EPIC-HR/SR studies, nirmatrelvir resistance was reported in less than 1% of participants.^1,2^ However, the frequency of nirmatrelvir resistance within community settings remains unclear, especially as these mutations reported in in vitro studies, in unique clinical cases, and in clinical trials may not reflect what is observed in the general population. Viral evolution of SARS-CoV-2 in the absence of immunosuppression or antiviral treatment could still lead to the development of resistance mutations, as evidenced by previous research on naturally occurring antiviral resistance mutations and by the 3.2% of untreated individuals with emergent nirmatrelvir resistance mutations in our analysis, which are most likely due to random mutations in the viral quasispecies.^5,6,9^

Overall, we found the frequency of emerging resistance to be higher in those who received nirmatrelvir, although we found that susceptibility to antiviral resistance was dependent on immune status, with the risk of emergent nirmatrelvir resistance mutations greater in treated, immunosuppressed participants. These results are concordant with our previous reports that immunosuppressed individuals treated with monoclonal antibodies have significantly greater risk of resistance emergence.^23^ These findings are likely due to a combination of factors, including the greater viral genetic diversity and prolonged duration of active viral replication in the setting of a suboptimal immune response.^23^ Additional studies are needed to assess whether combination antiviral therapy may be effective in enhancing viral clearance and preventing emergent drug resistance in the immunosuppressed population, as suggested by nonhuman primate models.^26^

In our study, the emerging antiviral resistance mutations detected were generally present only at low frequencies and were transient. These findings contrast with what has previously been reported with monoclonal antibody therapy. In our prior analysis of bamlanivimab-treated participants in the ACTIV-2 study, treatment-emergent resistance mutations rapidly increased in frequency to become the dominant variant over time.^15^ This difference is likely due to a few factors, including the extremely short half-life of nirmatrelvir compared with that of monoclonal antibodies (6 hours vs 17.6 days).^1,15^ The nirmatrelvir resistance mutations we observed may also confer a greater viral replicative fitness loss compared with the spike mutations that were reported in the monoclonal antibody study.^15,27^ Additionally, some of the nirmatrelvir resistance mutations we detected have been reported to need a compensatory mutation to overcome their fitness cost, such as E166V+L50F, but these compensatory mutations were not observed in our analysis.^4,27^ The low frequencies and transient nature of these mutations suggest that selection of these resistant variants and their rapid spread through the general population is unlikely given current variants and antiviral use practices. Our analysis of SARS-CoV-2 sequences in GISAID from before and after the FDA EUA for nirmatrelvir supported this idea, showing only a minimal increase in the prevalence of 1 nirmatrelvir resistance mutation, E166V, with a peak of approximately 1 in 10 000 sequences that was not sustained.

We and others have previously demonstrated that virologic rebound after nirmatrelvir can be detected in a substantial subset of participants,^12,13,28^ but the underlying cause of virologic rebound still remains unclear. In the 3 individuals who experienced posttreatment virologic rebound and had a nirmatrelvir resistance mutation emerge, all 3 mutations that were detected (E166V, P252L, and V297A) were present at less than 2% of the viral population and only at a single time point before reverting to the wild type. These mutations were detected at different points along the participants’ rebounding courses, with P252L and V297A emerging in 2 participants before the viral load started to rebound, while E166V emerged in the other participant at the peak viral load time point of the rebound. Even though E166V was detected when the participant’s viral load was the highest, the lack of a subsequent sustained increase in viral load suggests that this mutation did not confer significant resistance to nirmatrelvir. Additionally, given these 3 mutations’ presence at only low frequencies and their transient nature, it is unlikely that emerging nirmatrelvir resistance is a substantial contributor to virologic rebound after nirmatrelvir treatment in this minor subset of participants.^12,13,14^

Similar to our findings with nirmatrelvir, the remdesivir mutations detected in the 2 immunosuppressed participants were also present at low frequencies and transient. Nevertheless, we did find an interesting emergent mutation, R197L, that was present across multiple time points at increasing frequencies and became the dominant amino acid briefly before reverting to the wild type. However, this mutation is at a position that has not been previously reported to impact remdesivir activity, despite being adjacent to a known inhibitory position in N198.^3,11^ R197L remains an interesting site for future analysis into potential RdRp structural impacts that could have inhibitory effects, potentially explaining why it was selected in our study.

### Limitations

This study has limitations. One limitation is that we did not use unique molecular identifiers (UMIs), which have been used to report on linked, minority mutations. While we did not use UMIs in our sequencing approach to identify low frequency mutations, we used other techniques for confirming accuracy of our variant calling (ie, using a control library made with known variant sequences spiked at known concentrations).

There were a few characteristics that differed between the treatment groups. As expected, nirmatrelvir-treated participants were older and more frequently immunosuppressed. Notably, none of the untreated immunosuppressed participants developed nirmatrelvir resistance, but a larger study is needed to confirm this pattern more robustly. Due to the challenges of sampling outpatients with COVID-19, we were only able to collect AN samples 3 times a week, and our estimates represent the lower bound for the frequency of resistance emergence as more frequent sampling would likely have increased the detection of mutations across the cohort.

While we primarily used AN swab samples in this outpatient study to analyze resistance emergence in the upper respiratory tract, one limitation is that we were unable to sample the lower respiratory tract using samples like broncoalveolar lavage fluid. It is possible that the lower respiratory tract can act as another reservoir for SARS-CoV-2 infection with different frequencies of resistance mutations.^29,30,31^ However, nasal swabs are a standard mode of SARS-CoV-2 sampling and were used in the phase 3 randomized clinical trials for nirmatrelvir.^2^ Any variants resistant to nirmatrelvir that are transmitted would need to be present in the upper respiratory tract. Additionally, we do not have data about the accumulated dosage of nirmatrelvir in the lower respiratory tract tissues, which could also impact the emergence of resistance mutations.

Finally, we were unable to evaluate the phenotypic effects of these low frequency resistance mutations on viral fitness. We can study this in the future using viral outgrowth assays.

## Conclusions

In this cohort study of 156 individuals with acute COVID-19, we observed that antiviral resistance mutations emerged at a higher frequency in those who received treatment than what had been previously reported using consensus sequencing, especially in participants who were immunosuppressed. However, these resistance mutations seemed unlikely to be a substantial contributor to virologic rebound following nirmatrelvir treatment, have not increased in frequency in the overall population, and did not appear to pose a significant risk with current variants and antiviral drug usage patterns.

## Notes

Supplement 1.
eMethods.
**eTable 1.** List of Race Options Available for Participants to Self-Report in EPIC Database **eTable 2.** List of Ethnicity Options Available for Participants to Self-Report in EPIC Database **eTable 3.** List of nsp5 Nirmatrelvir (NTV) Resistance Mutations of Interest Included in This Analysis **eTable 4.** List of nsp12 Remdesivir Resistance Mutations of Interest Included in This Analysis **eTable 5.** Demographic Characteristics of Participants in This Subset of the POSITIVES Cohort **eTable 6.** Immunosuppression Status and Antiviral Resistance Prevalence of Participants **eFigure 1.** Screening and Enrollment Diagram for Nirmatrelvir and Untreated Cohorts **eFigure 2.** Screening and Enrollment Diagram for Remdesivir Cohort **eFigure 3.** Prevalence of Emergent Nirmatrelvir (NTV) Resistance Mutations in Untreated and NTV-Treated Individuals **eFigure 4.** Heat Map Showing Distribution of nsp5 Polymorphisms in Untreated and Nirmatrelvir (NTV) Treated Individuals **eFigure 5.** Viral Load and Mutational Landscape Graphs for All the Participants With an Emergent Antiviral Resistance Mutation **eFigure 6.** Heat Map Showing Distribution of nsp12 Polymorphisms in Remdesivir (RDV) Treated Individuals

Supplement 2.
Data Sharing Statement
