# Supplementary material for: Emerging SARS-CoV-2 Resistance After Antiviral Treatment
Source: JAMA Netw Open. 2024 Sep 25;7(9):e2435431. doi: 10.1001/jamanetworkopen.2024.35431 (PMC11425144; doi:10.1001/jamanetworkopen.2024.35431)
Supplement: Supplement 1. — eMethods. eTable 1. List of Race Options Available for Participants to Self-Report in EPIC Database eTable 2. List of Ethnicity Options Available for Participants to Self-Report in EPIC Database eTable 3. List of nsp5 Nirmatrelvir (NTV) Resistance Mutations of Interest Included in This Analysis eTable 4. List of nsp12 Remdesivir Resistance Mutations of Interest Included in This Analysis eTable 5. Demographic Characteristics of Participants in This Subset of the POSITIVES Cohort eTable 6. Immunosuppression Status and Antiviral Resistance Prevalence of Participants eFigure 1. Screening and Enrollment Diagram for Nirmatrelvir and Untreated Cohorts eFigure 2. Screening and Enrollment Diagram for Remdesivir Cohort eFigure 3. Prevalence of Emergent Nirmatrelvir (NTV) Resistance Mutations in Untreated and NTV-Treated Individuals eFigure 4. Heat Map Showing Distribution of nsp5 Polymorphisms in Untreated and Nirmatrelvir (NTV) Treated Individuals eFigure 5. Viral Load and Mutational Landscape Graphs for All the Participants With an Emergent Antiviral Resistance Mutation eFigure 6. Heat Map Showing Distribution of nsp12 Polymorphisms in Remdesivir (RDV) Treated Individuals [file jamanetwopen-e2435431-s001.pdf]

Supplemental Online Content

Tamura TJ, Choudhary MC, Deo R, et al; POSITIVES Study Team. Emerging SARS-CoV-2 resistance after antiviral treatment. *JAMA Netw Open.* 2024;7(9):e2435431. doi:10.1001/jamanetworkopen.2024.35431

eMethods.

eTable 1. List of Race Options Available for Participants to Self-Report in EPIC Database

eTable 2. List of Ethnicity Options Available for Participants to Self-Report in EPIC Database

eTable 3. List of nsp5 Nirmatrelvir (NTV) Resistance Mutations of Interest Included in This Analysis

eTable 4. List of nsp12 Remdesivir Resistance Mutations of Interest Included in This Analysis

eTable 5. Demographic Characteristics of Participants in This Subset of the POSITIVES Cohort

eTable 6. Immunosuppression Status and Antiviral Resistance Prevalence of Participants

eFigure 1. Screening and Enrollment Diagram for Nirmatrelvir and Untreated Cohorts

eFigure 2. Screening and Enrollment Diagram for Remdesivir Cohort

eFigure 3. Prevalence of Emergent Nirmatrelvir (NTV) Resistance Mutations in Untreated and NTV-Treated Individuals

eFigure 4. Heat Map Showing Distribution of nsp5 Polymorphisms in Untreated and Nirmatrelvir (NTV) Treated Individuals

eFigure 5. Viral Load and Mutational Landscape Graphs for All the Participants With an Emergent Antiviral Resistance Mutation

eFigure 6. Heat Map Showing Distribution of nsp12 Polymorphisms in Remdesivir (RDV) Treated Individuals

This supplemental material has been provided by the authors to give readers additional information about their work.

© 2024 Tamura TJ et al. *JAMA Network Open.*

eMethods.

Study Design and Sample Collection

For the minimum follow-up time, participants in the nirmatrelvir treated and untreated groups were required to have collected a swab at least 11 days or more after their baseline diagnostic COVID-19 test to be eligible for this analysis. The maximum follow-up time was variable among participants. When possible, we asked participants who remained PCR positive after 6 swabs (approximately 14 days since baseline diagnostic test) to continue to collect swabs until they test negative by PCR twice, which is what we defined as persistently undetectable viral load. However, not all participants were willing/able to accommodate the extra swabs (e.g., 1027), in which case we used data from all swabs that were available, even if they did not meet the criteria for persistently undetectable viral load.

For race and ethnicity, we took the self-reported demographics and grouped them into categories as follows: Black or African American, White, Other, Unknown for race and Hispanic or Latino, Not Hispanic or Latino, Other/Unknown for ethnicity. Other for race includes American Indian or Alaska Native, Asian, and Native Hawaiian or Other Pacific Islander. Race and ethnicity were assessed in this cohort as general demographic information, as well as the possibility for future analysis with this cohort that might stratify viral kinetics and mutation emergence by race and ethnicity.

© 2024 Tamura TJ et al. *JAMA Network Open.*

eTable 1. List of Race Options Available for Participants to Self-Report in EPIC Database

American Indian/Alaska Native	Skykomish
Asian	Snohomish
Native Hawaiian or Other Pacific Islander	Snoqualmie
Black or African American	Squaxin Island
White	Steilacoom
American Indian	Stillaguamish
Alaska Native	Suquamish
Abenaki	Swinomish
Algonquian	Tulalip
Apache	Upper Skagit
Arapaho	Iowa Sac and Fox
Arikara	Missouri Sac and Fox
Assiniboine	Oklahoma Sac and Fox
Assiniboine Sioux	Big Cypress
Bannock	Brighton
Blackfeet	Florida Seminole
Brotherton	Hollywood Seminole
Burt Lake Band	Oklahoma Seminole
Caddo	San Manual
Cahuilla	Absentee Shawnee
California Tribes	Eastern Shawnee
Canadian and Latin American Indian	Battle Mountain
Catawba	Duckwater
Cayuse	Elko
Chehalis	Ely
Chemakuan	Goshute
Chemehuevi	Panamint
Cherokee	Ruby Valley
Cherokee Shawnee	Skull Valley
Cheyenne	South Fork Shoshone
Cheyenne-Arapaho	Te-Moak Western Shoshone
Chickahominy	Timbi-Sha Shoshone
Chickasaw	Washakie
Chinook	Wind River Shoshone
Chippewa	Yomba

© 2024 Tamura TJ et al. *JAMA Network Open.*

Chippewa Cree	Duck Valley
Chitimacha	Fallon
Choctaw	Fort McDermitt
Chumash	Blackfoot Sioux
Clear Lake	Brule Sioux
Coeur D'Alene	Cheyenne River Sioux
Coharie	Crow Creek Sioux
Colorado River	Dakota Sioux
Colville	Flandreau Santee
Comanche	Fort Peck
Coos, Lower Umpqua, Siuslaw	Lake Traverse Sioux
Coos	Lower Brule Sioux
Coquilles	Lower Sioux
Costanoan	Mdewakanton Sioux
Coushatta	Miniconjou
Cowlitz	Oglala Sioux
Cree	Pine Ridge Sioux
Creek	Pipestone Sioux
Croatan	Prairie Island Sioux
Crow	Prior Lake Sioux
Cupeno	Rosebud Sioux
Delaware	Sans Arc Sioux
Diegueno	Santee Sioux
Eastern Tribes	Sisseton-Wahpeton
Esselen	Sisseton Sioux
Fort Belknap	Spirit Lake Sioux
Fort Berthold	Standing Rock Sioux
Fort Mcdowell	Teton Sioux
Fort Hall	Two Kettle Sioux
Gabrieleno	Upper Sioux
Grand Ronde	Wahpekute Sioux
Gros Ventres	Wahpeton Sioux
Haliwa	Wazhaza Sioux
Hidatsa	Yankton Sioux
Hoopa	Yanktonai Sioux
Hoopa Extension	Ak-Chin
Houma	Gila Bend
Inaja-Cosmit	San Xavier

© 2024 Tamura TJ et al. *JAMA Network Open.*

Iowa	Sells
Iroquois	Cow Creek Umpqua
Juaneno	Allen Canyon
Kalispel	Uintah Ute
Karuk	Ute Mountain Ute
Kaw	Gay Head Wampanoag
Kickapoo	Mashpee Wampanoag
Kiowa	Alpine
Klallam	Carson
Klamath	Dresslerville
Konkow	Ho-chunk
Kootenai	Nebraska Winnebago
Lassik	Table Bluff
Long Island	Barrio Libre
Luiseno	Pascua Yaqui
Lumbee	Chukchansi
Lummi	Tachi
Maidu	Tule River
Makah	Cocopah
Maliseet	Havasupai
Mandan	Hualapai
Mattaponi	Maricopa
Menominee	Mohave
Miami	Quechan
Miccosukee	Yavapai
Micmac	Coast Yurok
Mission Indians	Alaska Indian
Miwok	Eskimo
Modoc	Aleut
Mohegan	Alaskan Athabascan
Mono	Southeast Alaska
Nanticoke	Ahtna
Narragansett	Alatna
Navajo	Alexander
Nez Perce	Allakaket
Nomalaki	Alanvik
Northwest Tribes	Anvik
Omaha	Arctic

© 2024 Tamura TJ et al. *JAMA Network Open.*

Oregon Athabaskan	Beaver
Osage	Birch Creek
Otoe-Missouria	Cantwell
Ottawa	Chalkyitsik
Paiute	Chickaloon
Pamunkey	Chistochina
Passamaquoddy	Chitina
Pawnee	Circle
Penobscot	Cook Inlet
Peoria	Copper Center
Pequot	Copper River
Pima	Dot Lake
Piscataway	Doyon
Pit River	Eagle
Pomo	Eklutna
Ponca	Evansville
Potawatomi	Fort Yukon
Powhatan	Gakona
Pueblo	Galena
Puget Sound Salish	Grayling
Quapaw	Gulkana
Quinault	Healy Lake
Rappahannock	Holy Cross
Reno-Sparks	Hughes
Round Valley	Huslia
Sac and Fox	Iliamna
Salinan	Kaltag
Salish	Kluti Kaah
Salish and Kootenai	Knik
Schaghticoke	Koyukuk
Scott Valley	Lake Minchumina
Seminole	Lime
Serrano	Mcgrath
Shasta	Manley Hot Springs
Shawnee	Mentasta Lake
Shinnecock	Minto
Shoalwater Bay	Nenana
Shoshone	Nikolai

© 2024 Tamura TJ et al. *JAMA Network Open.*

Shoshone Paiute	Ninilchik
Siletz	Nondalton
Sioux	Northway
Siuslaw	Nulato
Spokane	Pedro Bay
Stewart	Rampart
Stockbridge	Ruby
Susanville	Salamatof
Tohono O'Odham	Seldovia
Tolowa	Slana
Tonkawa	Shageluk
Tygh	Stevens
Umatilla	Stony River
Umpqua	Takotna
Ute	Tanacross
Wailaki	Tanaina
Walla-Walla	Tanana
Wampanoag	Tanana Chiefs
Warm Springs	Tazlina
Wascopum	Telida
Washoe	Tetlin
Wichita	Tok
Wind River	Tyonek
Winnebago	Venetie
Winnemucca	Wiseman
Wintun	Tlingit-Haida
Wiyot	Tsimshian
Yakama	Angoon
Yakama Cowlitz	Central Council of Tlingit and Haida Tribes
Yaqui	Chilkat
Yavapai Apache	Chilkoot
Yokuts	Craig
Yuchi	Douglas
Yuman	Haida
Yurok	Hoonah
Chiricahua	Hydaburg
Fort Sill Apache	Kake
Jicarilla Apache	Kasaan

© 2024 Tamura TJ et al. *JAMA Network Open.*

Lipan Apache	Kenaitze
Mescalero Apache	Ketchikan
Oklahoma Apache	Klawock
Payson Apache	Pelican
San Carlos Apache	Petersburg
White Mountain Apache	Saxman
Northern Arapaho	Sitka
Southern Arapaho	Tenakee Springs
Wind River Arapaho	Tlingit
Fort Peck Assiniboine Sioux	Wrangell
Oklahoma Cado	Yakutat
Agua Caliente Cahuilla	Metlakatla
Augustine	Greenland Eskimo
Cabazon	Inupiat Eskimo
Los Coyotes	Siberian Eskimo
Morongo	Yupik Eskimo
Santa Rosa Cahuilla	Ambler
Torres-Martinez	Anaktuvuk
Cahto	Anaktuvuk Pass
Chimariko	Arctic Slope Inupiat
Coast Miwok	Arctic Slope Corporation
Digger	Atqasuk
Kawaiisu	Barrow
Kern River	Bering Straits Inupiat
Mattole	Brevig Mission
Red Wood	Buckland
Santa Rosa	Chinik
Takelma	Council
Wappo	Deering
Yana	Elim
Yuki	Golovin
Canadian Indian	Inalik Diomede
Central American Indian	Inupiaq
French American Indian	Kaktovik
Mexican American Indian	Kawerak
South American Indian	Kiana
Spanish American Indian	Kivalina
Hoh	Kobuk

© 2024 Tamura TJ et al. *JAMA Network Open.*

Quileute	Kotzebue
Cherokee Alabama	Koyuk
Cherokees of Northeast Alabama	Kwiguk
Cherokees of Southeast Alabama	Mauneluk Inupiat
Eastern Cherokee	Nana Inupiat
Echota Cherokee	Noatak
Etowah Cherokee	Nome
Northern Cherokee	Noorvik
Tuscola	Nuiqsut
United Keetowah Band of Cherokee	Point Hope
Western Cherokee	Point Lay
Northern Cheyenne	Selawik
Southern Cheyenne	Shaktoolik
Eastern Chickahominy	Shishmaref
Western Chickahominy	Shungnak
Clatsop	Solomon
Columbia River Chinook	Teller
Kathlamet	Unalakleet
Upper Chinook	Wainwright
Wakiakum Chinook	Wales
Willapa Chinook	White Mountain
Wishram	White Mountain Inupiat
Bad River	Mary's Igloo
Bay Mills Chippewa	Gambell
Bois Forte	Savoonga
Burt Lake Chippewa	Siberian Yupik
Fond du Lac	Akiachak
Grand Portage	Akiak
Grand Traverse Band of Ottawa/Chippewa	Alakanuk
Keweenaw	Aleknagik
Lac Courte Oreilles	Andreafsky
Lac du Flambeau	Aniak
Lac Vieux Desert Chippewa	Atmautluak
Lake Superior	Bethel
Leech Lake	Bill Moore's Slough
Little Shell Chippewa	Bristol Bay Yupik
Mille Lacs	Calista Yupik

© 2024 Tamura TJ et al. *JAMA Network Open.*

Minnesota Chippewa	Chefornak
Ontonagon	Chevak
Red Cliff Chippewa	Chuathbaluk
Red Lake Chippewa	Clark's Point
Saginaw Chippewa	Crooked Creek
St. Croix Chippewa	Dillingham
Sault Ste. Marie Chippewa	Eek
Sokoagon Chippewa	Ekuk
Turtle Mountain	Ekwok
White Earth	Emmonak
Rocky Boy's Chippewa Cree	Goodnews Bay
Clifton Choctaw	Hooper Bay
Jena Choctaw	Iqurmuit (Russian Mission)
Mississippi Choctaw	Kalskag
Mowa Band of Choctaw	Kasigluk
Oklahoma Choctaw	Kipnuk
Santa Ynez	Koliganek
Oklahoma Comanche	Kongiganak
Alabama Coushatta	Kotlik
Alabama Creek	Kwethluk
Alabama Quassarte	Kwigillingok
Eastern Creek	Levelock
Eastern Muscogee	Lower Kalskag
Kialegee	Manokotak
Lower Muscogee	Marshall
Machis Lower Creek Indian	Mekoryuk
Poarch Band	Mountain Village
Principal Creek Indian Nation	Naknek
Star Clan of Muscogee Creeks	Napaumute
Thlopthlocco	Napakiak
Tuckabachee	Napaskiak
Agua Caliente	Newhalen
Eastern Delaware	New Stuyahok
Lenni-Lenape	Newtok
Munsee	Nightmute
Oklahoma Delaware	Nunapitchukv
Rampough Mountain	Oscarville
Sand Hill	Pilot Station

© 2024 Tamura TJ et al. *JAMA Network Open.*

Campo	Pitkas Point
Capitan Grande	Platinum
Cuyapaipe	Portage Creek
La Posta	Quinhagak
Manzanita	Red Devil
Mesa Grande	St. Michael
San Pasqual	Scammon Bay
Santa Ysabel	Sheldon's Point
Sycuan	Sleetmute
Attacapa	Stebbins
Biloxi	Togiak
Georgetown (Eastern Tribes)	Toksook
Moor	Tulukskak
Nansemond	Tuntutuliak
Natchez	Tununak
Nausu Waiwash	Twin Hills
Nipmuc	Georgetown (Yupik-Eskimo)
Paugussett	St. Mary's
Pocomoke Acohonock	Umkumiate
Southeastern Indians	Alutiiq Aleut
Susquehanock	Bristol Bay Aleut
Tunica Biloxi	Chugach Aleut
Waccamaw-Siousan	Eyak
Wicomico	Koniag Aleut
Atsina	Sugpiaq
Trinity	Suqpigaq
Whilkut	Unangan Aleut
Iowa of Kansas-Nebraska	Tatitlek
Iowa of Oklahoma	Ugashik
Cayuga	Chignik
Mohawk	Chignik Lake
Oneida	Egegik
Onondaga	Igiugig
Seneca	Ivanof Bay
Seneca Nation	King Salmon
Seneca-Cayuga	Kokhanok
Tonawanda Seneca	Perryville
Tuscarora	Pilot Point

© 2024 Tamura TJ et al. *JAMA Network Open.*

Wyandotte	Port Heiden
Oklahoma Kickapoo	Chenega
Texas Kickapoo	Chugach Corporation
Oklahoma Kiowa	English Bay
Jamestown	Port Graham
Lower Elwha	Akhiok
Port Gamble Klallam	Agdaagux
Matinecock	Karluk
Montauk	Kodiak
Poospatuck	Larsen Bay
Setauket	Old Harbor
La Jolla	Ouzinkie
Pala	Port Lions
Pauma	Akutan
Pechanga	Aleut Corporation
Soboba	Aleutian
Twenty-Nine Palms	Aleutian Islander
Temecula	Atka
Mountain Maidu	Belkofski
Nishinam	Chignik Lagoon
Illinois Miami	King Cove
Indiana Miami	False Pass
Oklahoma Miami	Nelson Lagoon
Aroostook	Nikolski
Alamo Navajo	Pauloff Harbor
Canoncito Navajo	Qagan Toyagungin
Ramah Navajo	Qawalangin
Alsea	St. George
Celilo	St. Paul
Columbia	Sand Point
Kalapuya	South Naknek
Molala	Unalaska
Talakamish	Unga
Tenino	Asian Indian
Tillamook	Bangladeshi
Wenatchee	Bhutanese
Yahooskin	Burmese
Burt Lake Ottawa	Cambodian

© 2024 Tamura TJ et al. *JAMA Network Open.*

Michigan Ottawa	Chinese
Oklahoma Ottawa	Taiwanese
Bishop	Filipino
Bridgeport	Hmong
Burns Paiute	Indonesian
Cedarville	Japanese
Fort Bidwell	Korean
Fort Independence	Laotian
Kaibab	Malaysian
Las Vegas	Okinawan
Lone Pine	Pakistani
Lovelock	Sri Lankan
Malheur Paiute	Thai
Moapa	Vietnamese
Northern Paiute	Iwo Jiman
Owens Valley	Maldivian
Pyramid Lake	Nepalese
San Juan Southern Paiute	Singaporean
Southern Paiute	Madagascar
Summit Lake	Black
Utu Utu Gwaitu Paiute	African American
Walker River	African
Yerington Paiute	Bahamian
Indian Township	Barbadian
Pleasant Point Passamaquoddy	Dominican
Oklahoma Pawnee	Dominica Islander
Oklahoma Peoria	Haitian
Marshantucket Pequot	Jamaican
Gila River Pima-Maricopa	Tobagoan
Salt River Pima-Maricopa	Trinidadian
Central Pomo	West Indian
Dry Creek	Botswanan
Eastern Pomo	Ethiopian
Kashia	Liberian
Northern Pomo	Namibian
Scotts Valley	Nigerian
Stonyford	Zairean
Sulphur Bank	Polynesian

© 2024 Tamura TJ et al. *JAMA Network Open.*

Nebraska Ponca	Micronesian
Oklahoma Ponca	Melanesian
Citizen Band Potawatomi	Other Pacific Islander
Forest County	Native Hawaiian
Hannahville	Samoan
Huron Potawatomi	Tahitian
Pokagon Potawatomi	Tongan
Prairie Band	Tokelauan
Wisconsin Potawatomi	Guamanian or Chamorro
Acoma	Guamanian
Arizona Tewa	Chamorro
Cochiti	Mariana Islander
Hopi	Marshallese
Isleta	Palauan
Jemez	Carolinian
Keres	Kosraean
Laguna	Pohnpeian
Nambe	Saipanese
Picuris	Kiribati
Piro	Chuukese
Pojoaque	Yapese
San Felipe	Fijian
San Ildefonso	Papua New Guinean
San Juan Pueblo	Solomon Islander
San Juan De	New Hebrides
San Juan	European
Sandia	Middle Eastern or North African
Santa Ana	Arab
Santa Clara	Armenian
Santo Domingo	English
Taos	French
Tesuque	German
Tewa	Irish
Tigua	Italian
Zia	Polish
Zuni	Scottish
Duwamish	Assyrian
Kikiallus	Egyptian

© 2024 Tamura TJ et al. *JAMA Network Open.*

Lower Skagit	Iranian
Muckleshoot	Iraqi
Nisqually	Lebanese
Nooksack	Palestinian
Port Madison	Syrian
Puyallup	Afghanistani
Samish	Israeili
Sauk-Suiattle	Other Race
Skokomish	Unknown

© 2024 Tamura TJ et al. *JAMA Network Open.*

eTable 2. List of Ethnicity Options Available for Participants to Self-Report in EPIC Database

Hispanic or Latino	Canarian	Chilean
Not Hispanic or Latino	Spanish Basque	Colombian
Spaniard	Mexican American	Ecuadorian
Mexican	Mexicano	Paraguayan
Central American	Chicano	Peruvian
South American	La Raza	Uruguayan
Latin American	Mexican American Indian	Venezuelan
Puerto Rican	Costa Rican	South American Indian
Cuban	Guatemalan	Criollo
Dominican	Honduran	Unknown
Andalusian	Nicaraguan	
Asturian	Panamanian	
Castillian	Salvadoran	
Catalonian	Central American Indian	
Belearic Islander	Canal Zone	
Gallego	Argentinean	
Valencian	Bolivian	

© 2024 Tamura TJ et al. *JAMA Network Open.*

eTable 3. List of *nsp5* Nirmatrelvir (NTV) Resistance Mutations of Interest Included in This Analysis These mutations were compiled from the Stanford Coronavirus Antiviral & Resistance Database. Mutations were included if they had a reduction in susceptibility to NTV of at least 2.5-fold.

M49del	L167F	Q192G
G138S	P168del	Q192H
F140L	H172L	Q192I
F140S	H172N	Q192K
N142L	H172Q	Q192L
G143S	H172Y	Q192N
S144A	A173T	Q192P
S144E	A173V	Q192R
S144L	R188G	Q192S
S144P	R188S	Q192T
M165R	Q189I	Q192V
M165T	Q189K	Q192W
E166A	Q192A	Q192Y
E166G	Q192C	A194S
E166K	Q192D	P252L
E166Q	Q192E	V297A
E166V	Q192F	T304I

© 2024 Tamura TJ et al. *JAMA Network Open.*

eTable 4. List of *nsp12* Remdesivir Resistance Mutations of Interest Included in This Analysis These mutations were compiled from the Stanford Coronavirus Antiviral & Resistance Database, as well as the Food and Drug Administration's Prescribing Information document for RDV (indicated by asterisk). Mutations were included if they had a reduction in susceptibility to RDV of at least 2.5-fold.

E136V*
V166A*
N198S*
A376V
D484Y
S759A
V792I
M794I*
E796G
C799F
C799R
E802A
E802D

© 2024 Tamura TJ et al. *JAMA Network Open.*

eTable 5. Demographic Characteristics of Participants in This Subset of the POSITIVES Cohort

Abbreviations: NTV: nirmatrelvir, RDV: remdesivir

Characteristic, No. (%)	Untreated (n=63)	NTV Treated (n=79)	RDV Treated (n=14)
Age, median (IQR)	45 (33-62)	62 (51-71)	61 (54-70)
Sex			
Female	49 (77.8)	59 (74.7)	6 (42.9)
Male	14 (22.2)	20 (25.3)	8 (57.1)
Race			
Black/AA	6 (9.5)	10 (12.7)	2 (14.3)
White	43 (68.3)	57 (72.2)	10 (71.4)
Other	14 (22.2)	12 (15.2)	2 (14.3)
Ethnicity			
Hispanic/Latino	4 (6.3)	6 (7.6)	2 (14.3)
Non-Hispanic/Latino	49 (77.8)	67 (84.8)	10 (71.4)
Other/Unknown	10 (15.9)	6 (7.6)	2 (14.3)
COVID-19 Vaccines, median (IQR)	3 (3-4)	4 (3-5)	2 (1-3)
Days since last vaccine, median (IQR)	224 (163-325)	155 (83-248)	108 (54-149)
mAb[a] use	0 (0)	0 (0)	3 (21.4)
Immunosuppression[b]			
Absent	56 (88.9)	57 (72.2)	7 (50.0)
Present	7 (11.1)	22 (27.8)	7 (50.0)
SARS-CoV-2 Variant			
BA.2[c]	7 (11.1)	9 (11.4)	4 (28.6)
BA.5[d]	19 (30.2)	21 (26.6)	0 (0)
XBB[e]	20 (31.7)	30 (38.0)	2 (14.3)
Other	15 (23.8)	13 (16.4)	8 (57.1)
Not yet sequenced	2 (3.2)	6 (7.6)	0 (0)
Virologic rebound			
Absent	60 (95.2)	56 (70.9)	n/a
Present	3 (4.8)	23 (29.1)	n/a

[a]mAb: monoclonal antibody
[b] Immunosuppression was determined by clinicians who conducted a formal chart review in any participant who had an immunosuppressing condition or was taking an immunosuppressing medication
[c] BA.2 includes all BA.2 subvariants
[d] BA.5 includes all BA.5 subvariants
[e]XBB includes all XBB subvariants

© 2024 Tamura TJ et al. *JAMA Network Open.*

eTable 6. Immunosuppression Status and Antiviral Resistance Prevalence of Participants. Categorization of diagnoses and treatments for the immunosuppressed individuals in this study, including their antiviral treatment and their resistance mutation(s) that emerged.

ID	SARS-CoV-2 Antiviral Treatment	Diagnosis	Immunosuppressants/Treatment	Group	Resistance Mutation(s)
101	RDV	Heart and kidney transplant	Everolimus + prednisone 5mg daily + tacrolimus	S-HT	n/a
449	RDV	Granulomatosis with polyangiitis	Rituximab	S-A	n/a
459	RDV	Immune related adverse events, metastatic merkel cell carcinoma	Pembrolizumab, tacrolimus, prednisone, mycophenolate	S-HT	M794I
471	RDV	Marginal zone lymphoma (CR)	Obinutuzumab + cyclophosphamide	S-HT	n/a
475	NTV	Multiple sclerosis	Rituximab within 12 months of COVID-19	S-A	n/a
497	RDV	Diffuse large B-cell lymphoma (CR), hypogammaglobulinemia	Rituximab-cyclophosphamide	S-HT	n/a
531	NTV	Sarcoidosis	Infliximab	NS	n/a
547	Untreated	Rheumatoid arthritis	Tocilizumab + methotrexate	NS	n/a
551	NTV	Psoriatic arthritis	Infliximab	NS	n/a
552	NTV	Seronegative spondyloarthropathy	Adalimumab + methotrexate	NS	n/a
557	NTV	Rheumatoid arthritis, systemic lupus erythematosus	Methotrexate	NS	n/a
563	NTV	Rheumatoid arthritis	Adalimumab	NS	Q189K
569	NTV	Systemic lupus erythematosus	Hydroxychloroquine + methylprednisolone daily 6mg	NS	n/a
573	NTV	Inflammatory arthritis	Adalimumab + hydroxychloroquine	NS	n/a
597	NTV	Giant cell arteritis, polymyalgia rheumatica	Tocilizumab + prednisone orally 5mg daily	NS	Q189K
658	NTV	Rheumatoid arthritis	Tocilizumab	NS	n/a
687	NTV	Rheumatoid arthritis, systemic lupus erythematosus	Hydroxychloroquine, methotrexate	NS	n/a
735	Untreated	Psoriatic arthritis	Adalimumab	NS	n/a
768	NTV	Ankylosing spondylitis	Secukinumab	NS	Q189K

© 2024 Tamura TJ et al. *JAMA Network Open.*

ID	SARS-CoV-2 Antiviral Treatment	Diagnosis	Immunosuppressants/Treatment	Group	Resistance Mutation(s)
805	NTV	Ulcerative colitis, inflammatory arthritis	Golimumab, methotrexate	NS	n/a
870	RDV	Mantle cell lymphoma	Bendamustine and rituximab within 12 months	S-HT	N198S, V792I, M794I
945	NTV	IgG4 related disease	Rituximab	S-A	n/a
953	Untreated	Rheumatoid arthritis	Infliximab, methotrexate, prednisone 3mg	NS	n/a
1223	RDV	Acute myeloid leukemia, hematopoietic stem cell transplantation	Tacrolimus, prednisone 10mg, venatoclax and chemotherapy until 2022	S-HT	n/a
1235	NTV	Psoriatic arthritis	Methotrexate, etancercept	NS	n/a
1236	NTV	Systemic lupus erythematosus	Prednisone orally 5mg daily, methotrexate, hydroxychloroquine	NS	n/a
1355	NTV	Rheumatoid arthritis	Baricitinib	NS	n/a
1389	Untreated	Rheumatoid arthritis	Methotrexate, hydroxychloroquine, adalimumab	NS	n/a
1399	Untreated	Uveitis and retinal vasculitis	Methotrexate, infliximab	NS	n/a
1472	NTV	Mantle cell lymphoma	Rituximab, bendamustine	S-HT	n/a
1473	NTV	Breast cancer	Pertuzumab-trastuzumab-hyaluronidase-zzxf	NS	P252L
1504	Untreated	Breast cancer	Eribulin, fulvestrant, sacituzumab govitecan, pembrolizumab	NS	n/a
1521	NTV	Relapsing polychondritis	Azathioprine + adalimumab	NS	n/a
1556	NTV	Rheumatoid arthritis	Methotrexate, adalimumab (used etanercept in early 2023)	NS	n/a
1557	Untreated	Takayasu arteritis	Adalimumab	NS	n/a
1560	NTV	Rheumatoid arthritis	Infliximab, methotrexate	NS	V297A

S-HT: severe hematological oncology/transplant, S-A: severe autoimmune disease/B-cell deficient, NS: non-severe immunosuppression, CR: complete remission, RDV: remdesivir, NTV: nirmatrelvir

© 2024 Tamura TJ et al. *JAMA Network Open.*

eFigure 1. Screening and Enrollment Diagram for Nirmatrelvir and Untreated Cohorts

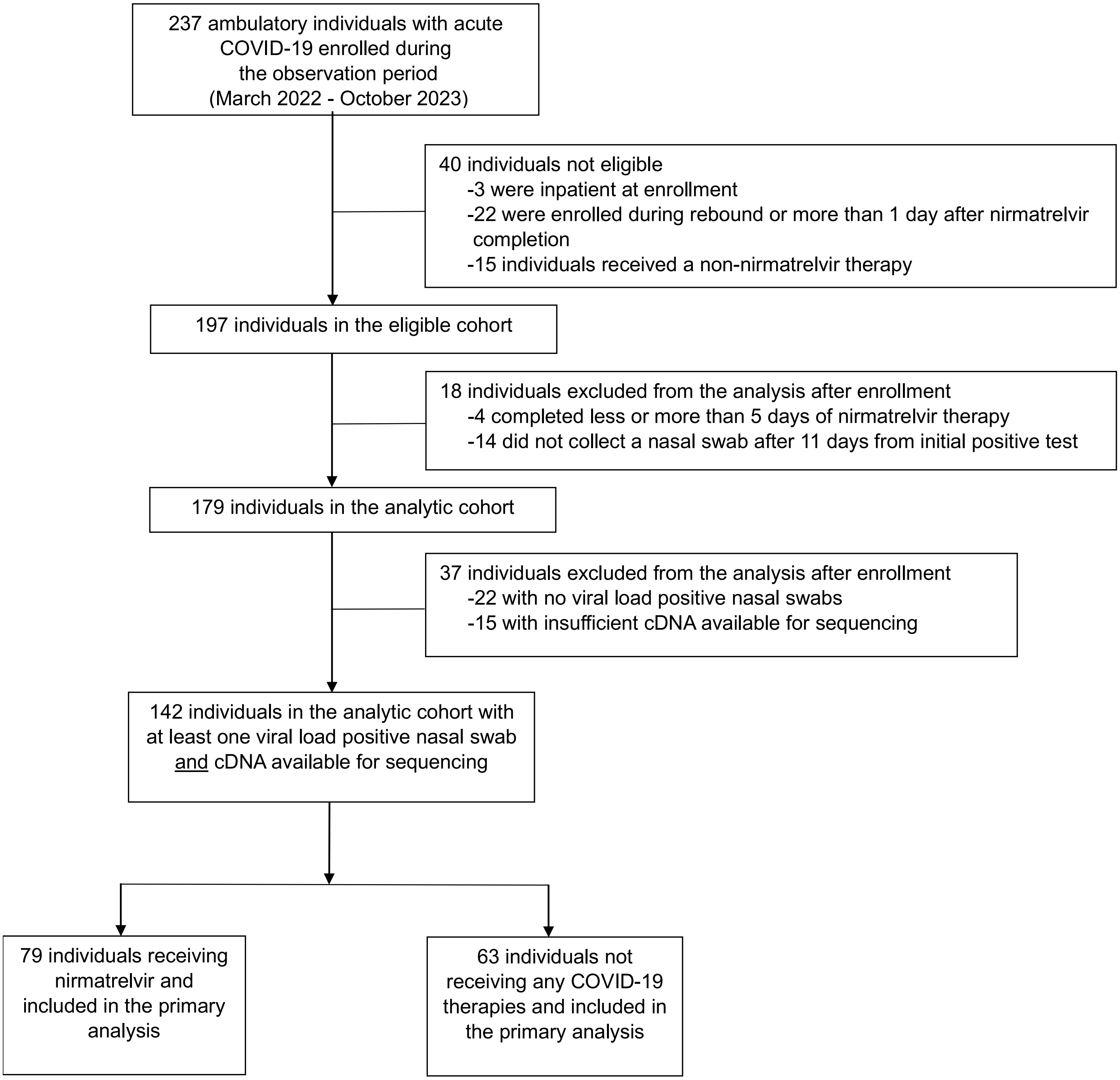

© 2024 Tamura TJ et al. *JAMA Network Open.*

eFigure 2. Screening and Enrollment Diagram for Remdesivir Cohort

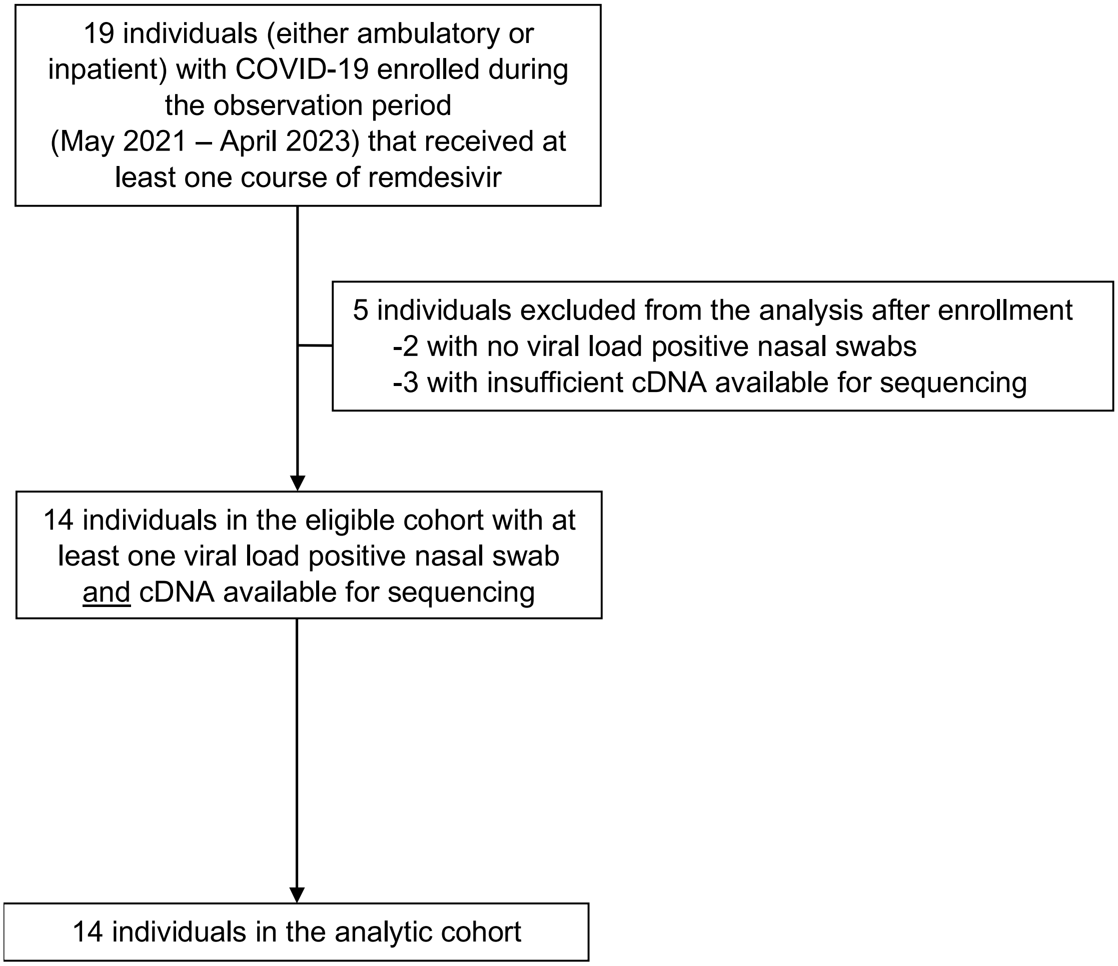

© 2024 Tamura TJ et al. *JAMA Network Open.*

eFigure 3. Prevalence of Emergent Nirmatrelvir (NTV) Resistance Mutations in Untreated and NTV-Treated Individuals. *P* value was calculated using a two-sided Boschloo's test.

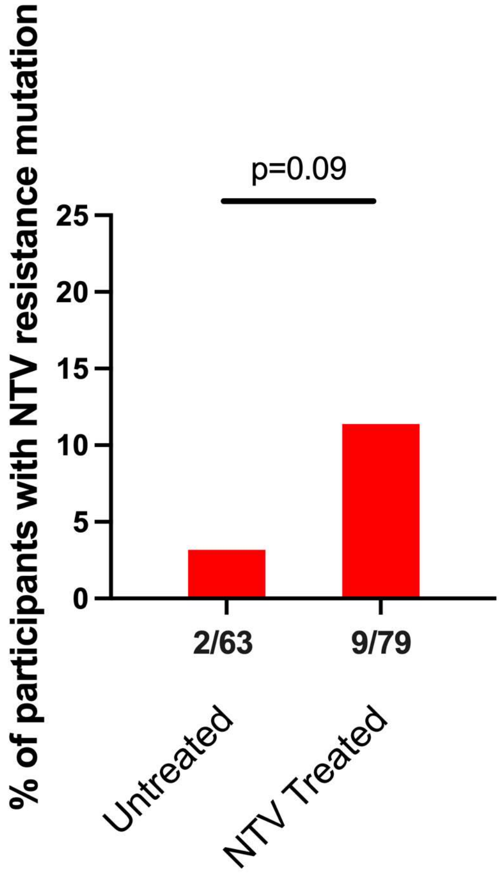

© 2024 Tamura TJ et al. *JAMA Network Open.*

eFigure 4. Heat Map Showing Distribution of *nsp5* Polymorphisms in Untreated and Nirmatrelvir (NTV) Treated Individuals. NTV treated individuals are shown below the red line and untreated individuals are shown above the red line. Each row represents the highest frequency polymorphism that was observed across multiple timepoints of an individual. The green bars highlight the NTV resistance mutations of interest that emerged. Using our control library, a calculated threshold was set for identifying low frequency mutations at 1% for viral loads ≥3 $\log_{10}$ SARS-CoV-2 RNA copies/mL and at 10% for viral loads <3 $\log_{10}$ SARS-CoV-2 RNA copies/mL.

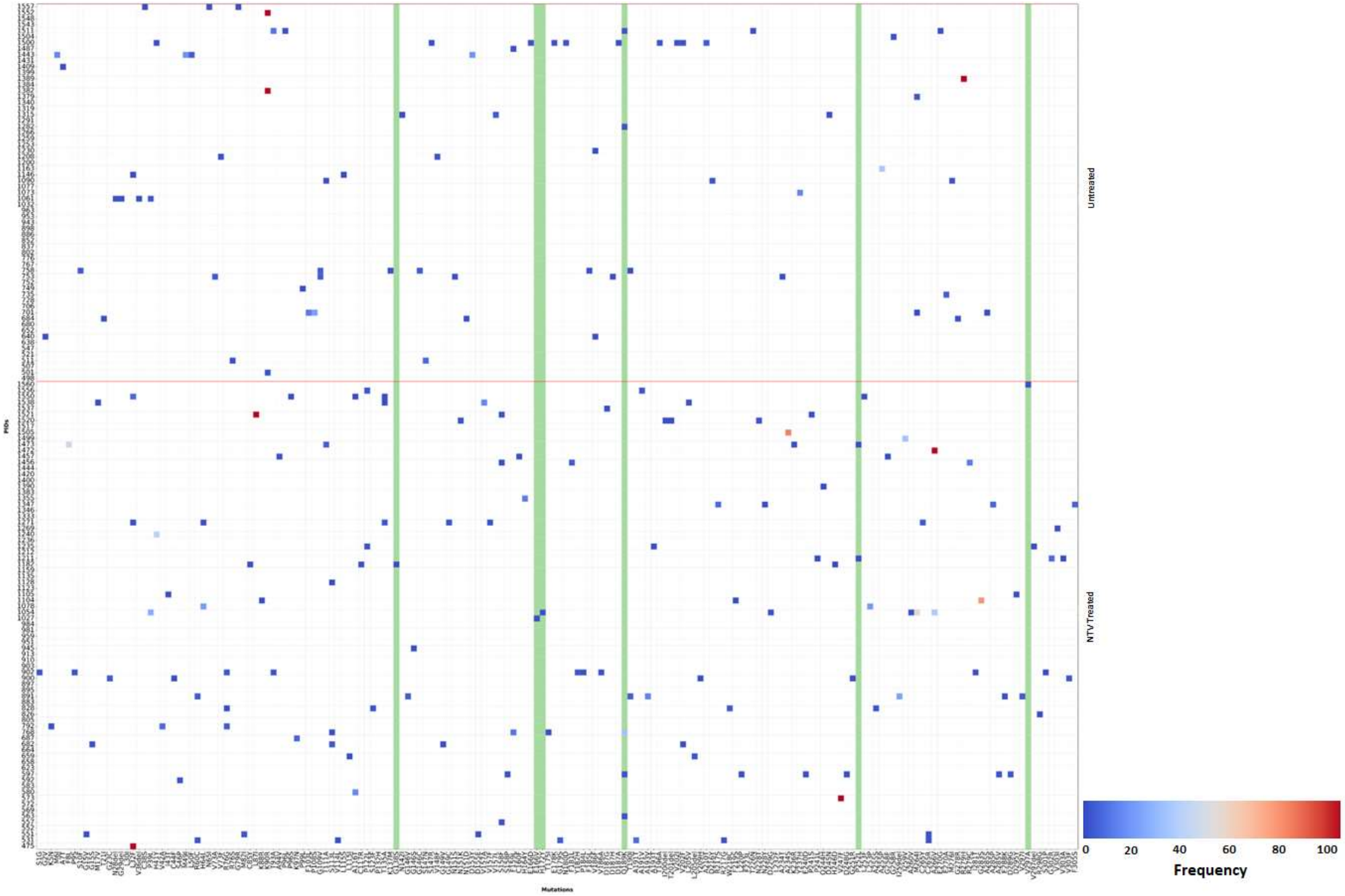

© 2024 Tamura TJ et al. *JAMA Network Open.*

eFigure 5. Viral Load and Mutational Landscape Graphs for All the Participants With an Emergent Antiviral Resistance Mutation. Abbreviations: IS: immunosuppressed, NTV: nirmatrelvir, RDV: remdesivir.

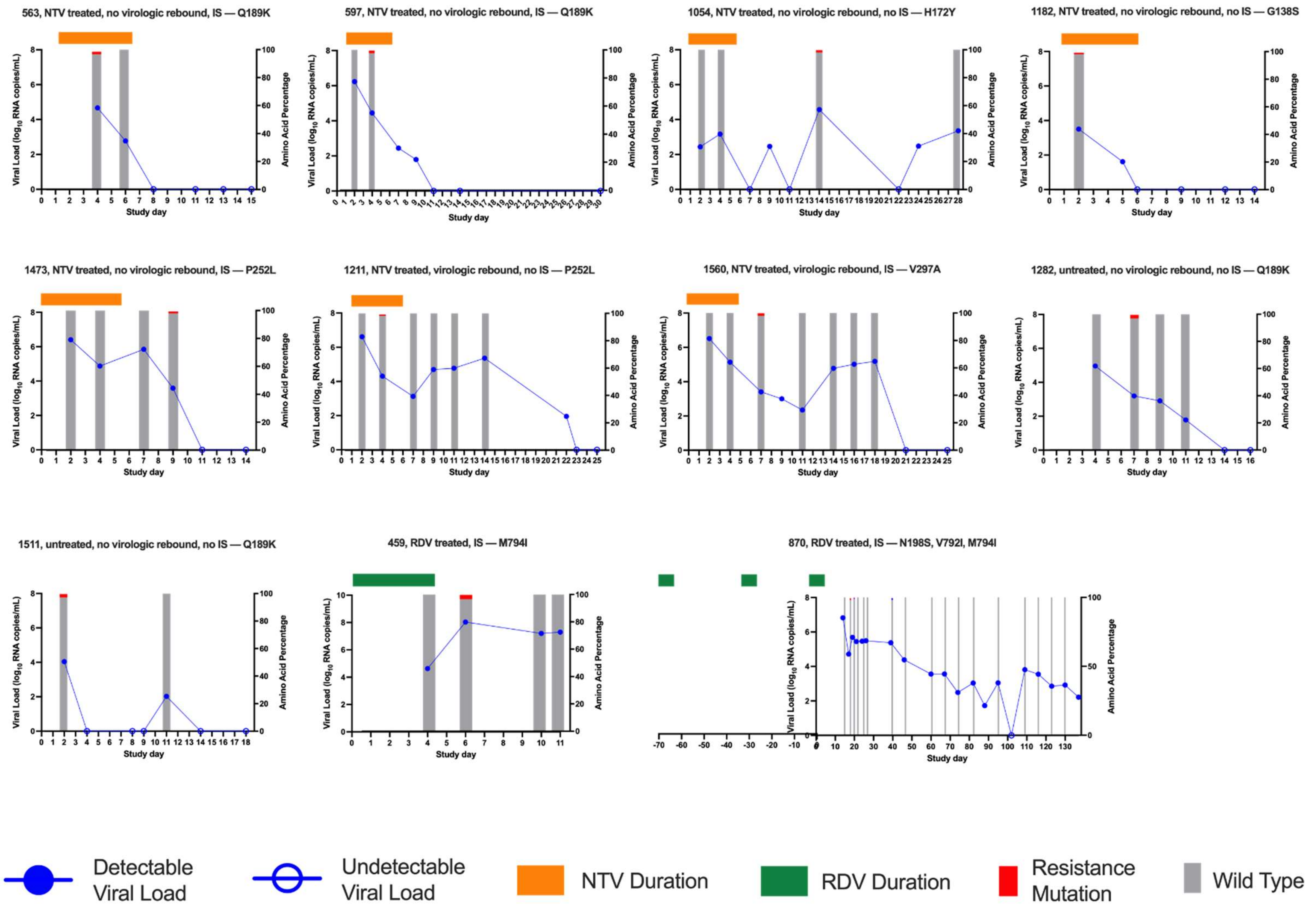

© 2024 Tamura TJ et al. *JAMA Network Open.*

eFigure 6. Heat Map Showing Distribution of *nsp12* Polymorphisms in Remdesivir (RDV) Treated Individuals. Each row represents the highest frequency polymorphism that was observed across multiple timepoints of an individual. The green bars highlight the RDV resistance mutations of interest that emerged. Using our control library, a calculated threshold was set for identifying low frequency mutations at 1% for viral loads ≥3 $\log_{10}$ SARS-CoV-2 RNA copies/mL and at 10% for viral loads <3 $\log_{10}$ SARS-CoV-2 RNA copies/mL.

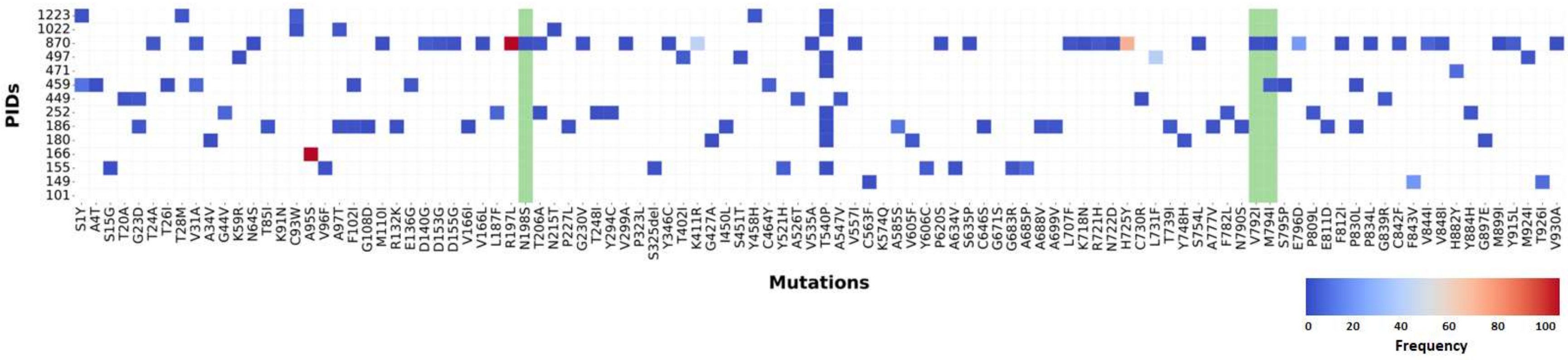

© 2024 Tamura TJ et al. *JAMA Network Open.*
