# Supplementary material for: Emerging SARS-CoV-2 Resistance After Antiviral Treatment
Source: JAMA Netw Open. 2024 Sep 25;7(9):e2435431. doi: 10.1001/jamanetworkopen.2024.35431 (PMC11425144; doi:10.1001/jamanetworkopen.2024.35431)
Supplement: Supplement 2. — Data Sharing Statement [file jamanetwopen-e2435431-s002.pdf]

Data Sharing Statement

Tamura. Emerging SARS-CoV-2 Resistance After Antiviral Treatment. *JAMA Netw Open*. Published September 25, 2024. doi:10.1001/jamanetworkopen.2024.35431

Data
Data available: Yes
Data types: Deidentified participant data, Data dictionary
How to access data: jli@bwh.harvard.edu
When available: With publication

Supporting Documents
Document types: None

Additional Information
Who can access the data: Anyone requesting the data
Types of analyses: For sequencing analysis
Mechanisms of data availability: With a signed data access agreement
